# Strain-Specific Systematic Review with Meta-Analysis of Probiotics Efficacy in the Treatment of Irritable Bowel Syndrome

**DOI:** 10.3390/jcm15031152

**Published:** 2026-02-02

**Authors:** Roman Maslennikov, Eva Gosteeva, Vera Ananeva, Lada Korshunova, Anastasya Kravtsowa, Elena Poluektova, Anatoly Ulyanin, Alexey Sigidaev, Patimat Kikhasurova, Vladimir Ivashkin

**Affiliations:** 1 Department of Internal Medicine, Gastroenterology and Hepatology, Sechenov University, Moscow 119435, Russiao.ladushek@mail.ru (L.K.); kikhasurova_p_m@student.sechenov.ru (P.K.);; 2 Department of Clinical Disciplines, Tyumen State Medical University, Tyumen 625023, Russia; tgmu@tyumsmu.ru

**Keywords:** probiotic, gut, abdominal pain, bloating, diarrhea, constipation, functional gut disease, microbiota, microbiome

## Abstract

**Background**: Many probiotic strains have been studied in relation to irritable bowel syndrome (IBS). The aim of this study was to identify probiotic strains demonstrating efficacy in the management of IBS based on meta-analyses of randomized placebo-controlled trials (RPCTs). **Methods**: This systematic review was registered in the PROSPERO database (CRD420251047092). Searches were conducted in PubMed and Scopus on 8 April 2025. Additional completed studies with available results were identified through ClinicalTrials.gov. An additional search of the Cochrane Central Register of Controlled Trials (CENTRAL), including records indexed in EMBASE, was conducted in December 2025 and did not identify any additional studies. RPCTs were included if they evaluated single-strain probiotics without additional active components compared with a placebo in patients with IBS. Studies whose results could not be meta-analyzed were excluded. **Results:** A total of 2643 records were identified; 32 articles evaluating 10 probiotic strains were included in the meta-analyses. Meta-analyses demonstrated the efficacy of *Bifidobacterium longum* (formerly *Bifidobacterium infantis*) 35624, *Lactobacillus rhamnosus* GG, *Lactiplantibacillus plantarum* 299v (DSM 9843), *Saccharomyces cerevisiae* CNCM I-3856, and *Bacillus coagulans* Unique IS2 (MTCC 5260) in improving key IBS symptoms. Meta-analyses also demonstrated that *Bacillus coagulans* MTCC 5856 improved quality of life for those with IBS. Conflicting results were observed for *Saccharomyces boulardii* CNCM I-745. Meta-analyses did not demonstrate the efficacy of *Escherichia coli* Nissle 1917, *Lactobacillus gasseri* BNR17, or *Lactobacillus casei* Shirota. **Conclusions:** Several probiotic strains demonstrated efficacy in the treatment of IBS in meta-analyses of RPCTs.

## 1. Introduction

Irritable bowel syndrome (IBS) is one of the most common gastrointestinal disorders worldwide [1]. Despite the wide range of pharmacological treatments proposed, their efficacy is often limited [1,2,3,4,5]. Given the established role of the gut microbiota in the pathogenesis of IBS [6,7,8], the use of live microorganisms (probiotics) has attracted considerable interest as a therapeutic approach. Numerous clinical trials have investigated the efficacy of probiotics in the management of IBS; however, their results have been inconsistent. To synthesize the available evidence, multiple systematic reviews with meta-analyses have been published [9,10,11,12,13,14,15,16,17,18,19,20,21,22,23,24,25,26,27,28,29,30,31,32,33,34,35,36,37,38] (Supplementary Table S1). While most of these reviews concluded that probiotics are generally effective in IBS, substantial heterogeneity was observed, suggesting strain-specific differences in therapeutic effects.

Importantly, the vast majority of published systematic reviews did not perform meta-analyses at the level of individual probiotic strains. The most recent strain-specific meta-analysis of probiotic efficacy in IBS was published more than four years ago [33] and therefore requires updating. Moreover, this analysis included studies evaluating multi-strain formulations [39,40,41,42,43,44,45,46,47,48,49,50], as well as probiotics combined with prebiotics [51] or simethicone [52], which precludes a reliable assessment of the true effects of individual probiotic strains.

In accordance with the principles of evidence-based medicine, meta-analyses of randomized placebo-controlled trials (RPCTs) provide the highest level of evidence, as they minimize bias and allow an assessment of the reproducibility of results. In this systematic review, we aimed to identify specific probiotic strains demonstrating reproducible benefits across different IBS outcomes based on meta-analyses of RPCTs.

## 2. Materials and Methods

**Figure 1:**
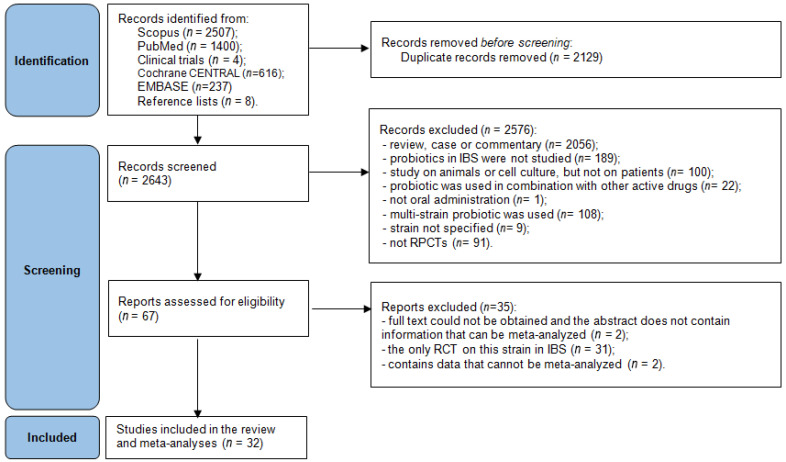
PRISMA flow diagram for the systematic review.

This systematic review was registered in the PROSPERO database (CRD420251047092) and conducted in accordance with the PRISMA guidelines [53]. On 8 April 2025, searches of the literature were performed in PubMed and Scopus using the search query “irritable AND bowel AND syndrome AND probiotic”. A total of 2507 records were identified in Scopus and 1400 in PubMed. After the removal of duplicates, the titles and abstracts of 2635 publications were screened. Additional completed studies with available results were identified through ClinicalTrials.gov using the condition “irritable bowel syndrome” and the keyword “probiotic”, with the filter “results posted”. The reference lists of all identified meta-analyses were also screened, yielding eight additional eligible studies. In total, 2643 records were assessed (Figure 1).

Additional searches were performed using the following terms: “irritable AND bowel AND syndrome AND Bacillus”, “irritable AND bowel AND syndrome AND Streptococcus”, “irritable AND bowel AND syndrome AND Lactobacillus”, “irritable AND bowel AND syndrome AND Bifidobacterium”, “irritable AND bowel AND syndrome AND Saccharomyces”, and “(probiotic[MeSH Terms]) AND (irritable bowel syndrome[MeSH Terms])”. These searches did not identify any additional eligible studies. An additional search of the Cochrane Central Register of Controlled Trials (CENTRAL) was conducted using the term “irritable bowel syndrome probiotic”. As of December 2025, 616 trials were identified, including 237 records indexed in EMBASE. No new relevant studies with available results were identified for inclusion. Searches using generic probiotic strain names in this database did not increase the number of eligible studies.

Publications that did not report clinical trials evaluating the efficacy of orally administered probiotics in human patients with IBS were excluded. Specifically, reviews, systematic reviews, editorials, case reports, commentaries, corrections, animal or in vitro studies, studies involving non-viable bacteria (metabiotics), and studies evaluating non-oral routes of probiotic administration were excluded. Studies were also excluded if they lacked a placebo control, evaluated probiotics in combination with other active treatments, did not specify the probiotic strain, or investigated multi-strain probiotic formulations.

Subsequently, the number of eligible publications available for each probiotic strain was assessed. Strains supported by only a single eligible publication were excluded. The final analysis included only probiotic strains for which efficacy in IBS was evaluated in more than one randomized placebo-controlled trial (RPCT).

No language restrictions were applied.

Study selection was performed independently by two reviewers (R.M. and one of the co-authors: E.G., A.K., L.K., or V.A.). Any disagreements were resolved by consensus; if consensus could not be reached, the decision of the principal investigator (V.I.) was considered final. The authors attempted to obtain full-text versions of all potentially eligible studies. When necessary, corresponding authors were contacted by email or via the scientific social network ResearchGate. Studies were excluded if the full text could not be obtained and if the data reported in the abstract were insufficient for meta-analysis.

Data were extracted from all included studies using a predefined data extraction form. Extracted variables included country of origin, probiotic strain, daily dose, treatment duration, sample size in each study arm, IBS subtype, diagnostic criteria, and patient demographics (age and sex). Whenever possible, data were extracted specifically for participants included in the final analyses of the original studies; otherwise, data reported at baseline or study inclusion were used.

Outcome data were also extracted. The primary outcomes were a reduction in abdominal pain, bloating, and other IBS-related symptoms, as well as an improvement in quality of life and overall IBS symptom severity. The secondary outcome was the incidence of adverse events. Data extraction was performed independently by two reviewers (R.M. and one of the co-authors: E.G., A.K., L.K., or V.A.). Discrepancies were resolved by consensus, with arbitration by the principal investigator (V.I.) when necessary.

The risk of bias of the included studies was assessed using the Cochrane Risk of Bias 2 (RoB 2) tool [54]. Assessment was performed independently by two reviewers (R.M. and one of the co-authors: E.G., A.K., L.K., or V.A.). Discrepancies were resolved by consensus, and if consensus could not be reached, the decision of the principal investigator (V.I.) was considered final.

The primary outcomes were the standardized mean differences in changes from baseline in symptom rating scales, stool frequency, and stool consistency according to the Bristol Stool Scale. Additionally, the odds ratio (OR) for clinically significant symptom improvement with probiotics versus placebo was analyzed. Visual data were digitized using Screen Calipers 4.0 (Iconico, USA). Standard errors of the mean were converted to standard deviations by multiplying by the square root of the sample size. Statistical heterogeneity was assessed using the I^2^ statistic. Values of I^2^ < 25% were considered indicative of low heterogeneity, whereas values > 75% indicated high heterogeneity. A random-effects model was applied in cases of high heterogeneity (I^2^ > 75%) and a fixed-effects model was used otherwise. Meta-analysis results were presented as forest plots. When a meta-analysis included more than four studies, publication bias was evaluated using funnel plots and the Begg–Mazumdar rank correlation test. All calculations, meta-analyses, and publication bias assessments were performed using the Comprehensive Meta-Analysis software (version 3.3.0.70).

This study was conducted without financial support, and there are no conflicts of interest. The detailed study protocol has not been published. Additional data can be obtained from the corresponding author by e-mail upon reasonable request.

## 3. Results

Among the 2643 studies reviewed, 67 RPCTs assessed the efficacy of oral single-strain probiotics in IBS, with the specific strain indicated. Of these, 10 strains were investigated in at least 2 studies to meta-analyze, with a total of 32 studies describing their efficacy. These studies were included in this systematic review. The characteristics of these studies are presented in Table 1, Table 2 and Table 3.

### 3.1. Bifidobacterium longum (Bifidobacterium infantis) 35624

**Figure 2:**
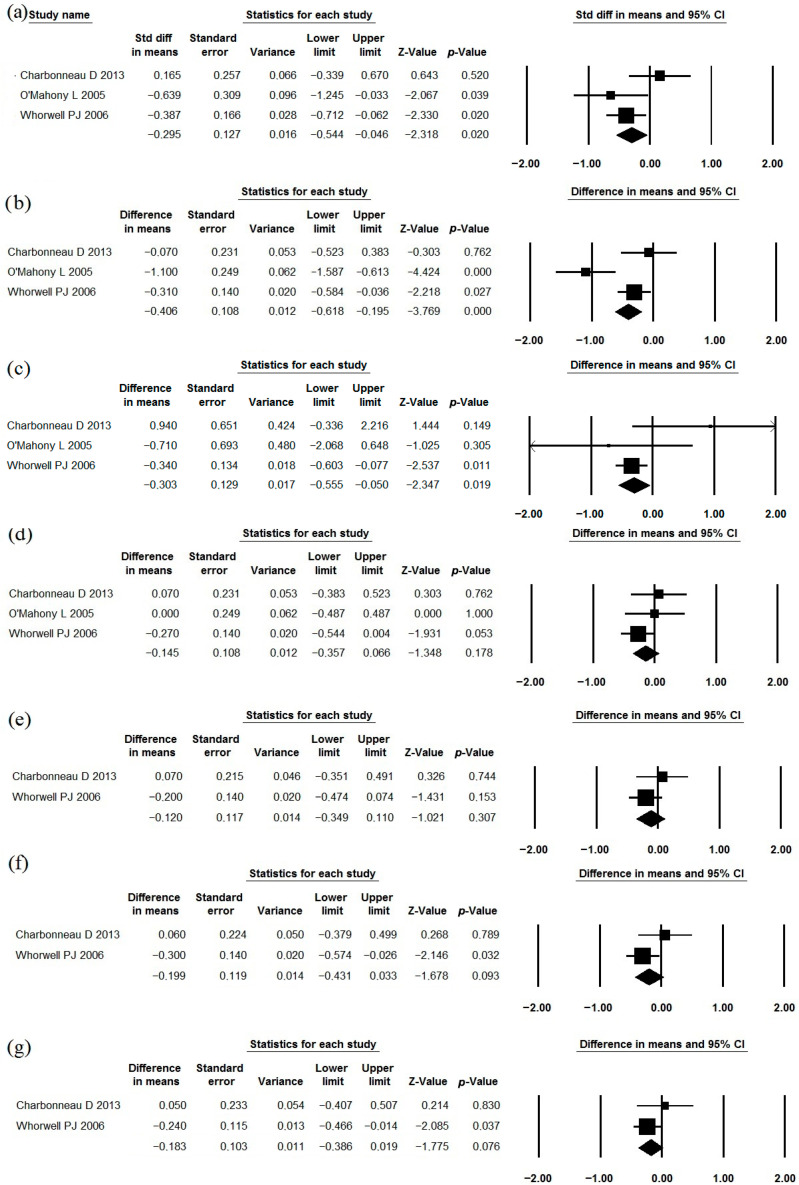
Meta-analysis of studies on the effect of *Bifidobacterium longum* 35624 on the severity of abdominal pain (**a**), straining/difficulty during defecation (**b**), symptoms in general (**c**), bloating (**d**), urgency (**e**), a feeling of incomplete evacuation (**f**), and passage of gas (**g**) in irritable bowel syndrome.

The use of *Bifidobacterium longum* (*Bifidobacterium infantis*) 35624 as a single strain has been studied in the treatment of IBS in three studies [55,56,57] that met the inclusion criteria (Table 1, Table 2 and Table 3). A meta-analysis of their results demonstrated that this strain effectively reduces the severity of abdominal pain, straining/difficulty during defecation, and IBS symptoms in general (Figure 2). However, its effect on bloating, urgency, a feeling of incomplete evacuation, and passage of gas was insufficient (Figure 2). The heterogeneity of the study results varied significantly (I^2^ = 57.9%, 10.4%, 50.8%, 20.6%, 10.3%, 46.3%, 19.5%, respectively). Indicators of quality of life were reported in a manner that did not allow for meta-analysis. The incidence of adverse effects in the probiotic groups did not differ significantly from that in the placebo groups (Table 2).

### 3.2. Lactobacillus rhamnosus (Lacticaseibacillus rhamnosus; Lactobacillus casei subsp. rhamnosus) GG

**Figure 3:**
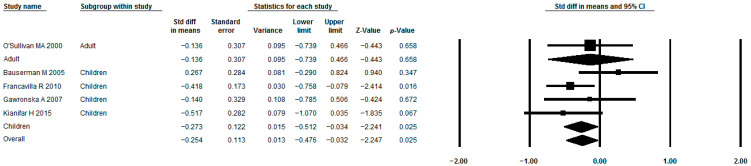
Meta-analysis of studies on the effect of *Lactobacillus rhamnosus* GG on the severity of abdominal pain in irritable bowel syndrome.

**Figure 4:**
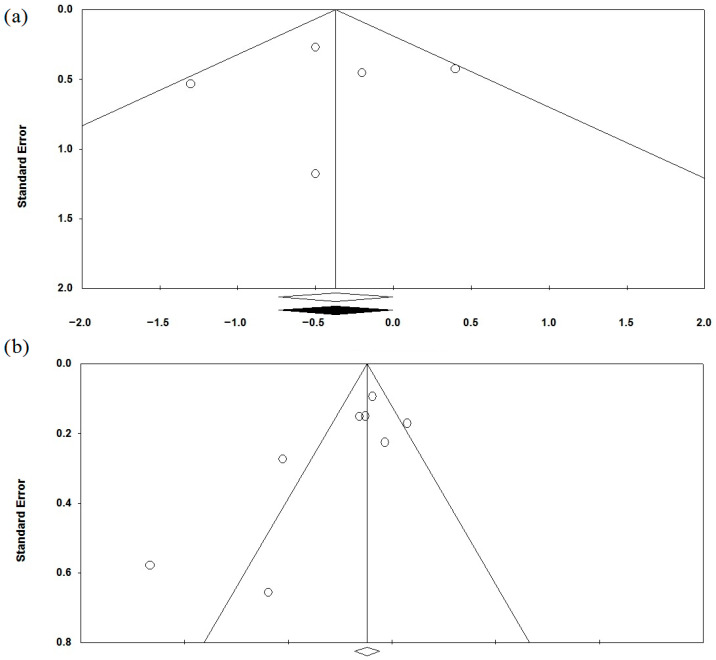
Funnel plots for assessment of publication bias in a meta-analysis of the results of a study on the efficacy of *Lactobacillus rhamnosus* GG (**a**) and *Saccharomyces cerevisiae* CNCM I-3856 (**b**) in the treatment of abdominal pain in IBS.

The use of *Lactobacillus rhamnosus* (*Lacticaseibacillus rhamnosus*; *Lactobacillus casei* subsp. *rhamnosus*) GG as a single strain has been studied in the treatment of IBS in five studies [58,59,60,61,62] that met the criteria (Table 1, Table 2 and Table 3). This probiotic significantly reduced pain (Figure 3). The heterogeneity of study results was moderate (I^2^ = 25.9%). When only studies in children were considered, the positive effect was maintained (I^2^ = 42.7%). However, the beneficial effect was lost across all subgroups in a meta-analysis based on the diagnostic criteria used for IBS (Supplementary Figure S1a). In a subgroup analysis by drug dose, a beneficial effect was observed with a daily probiotic dose of 6 billion cells, but not with 20 or 40 billion cells (Supplementary Figure S1b). In a subgroup analysis by treatment duration, benefits were seen only with 8 weeks of treatment, but not with 4 or 6 weeks (Supplementary Figure S1c). Other IBS symptoms and quality of life indicators could not be meta-analyzed from these studies. Most studies reported no adverse effects with this strain (Table 2). No publication bias was detected (*p* = 0.807) (Figure 4a).

### 3.3. Lactiplantibacillus plantarum (Lactobacillus plantarum) 299v (DSM 9843)

**Figure 5:**
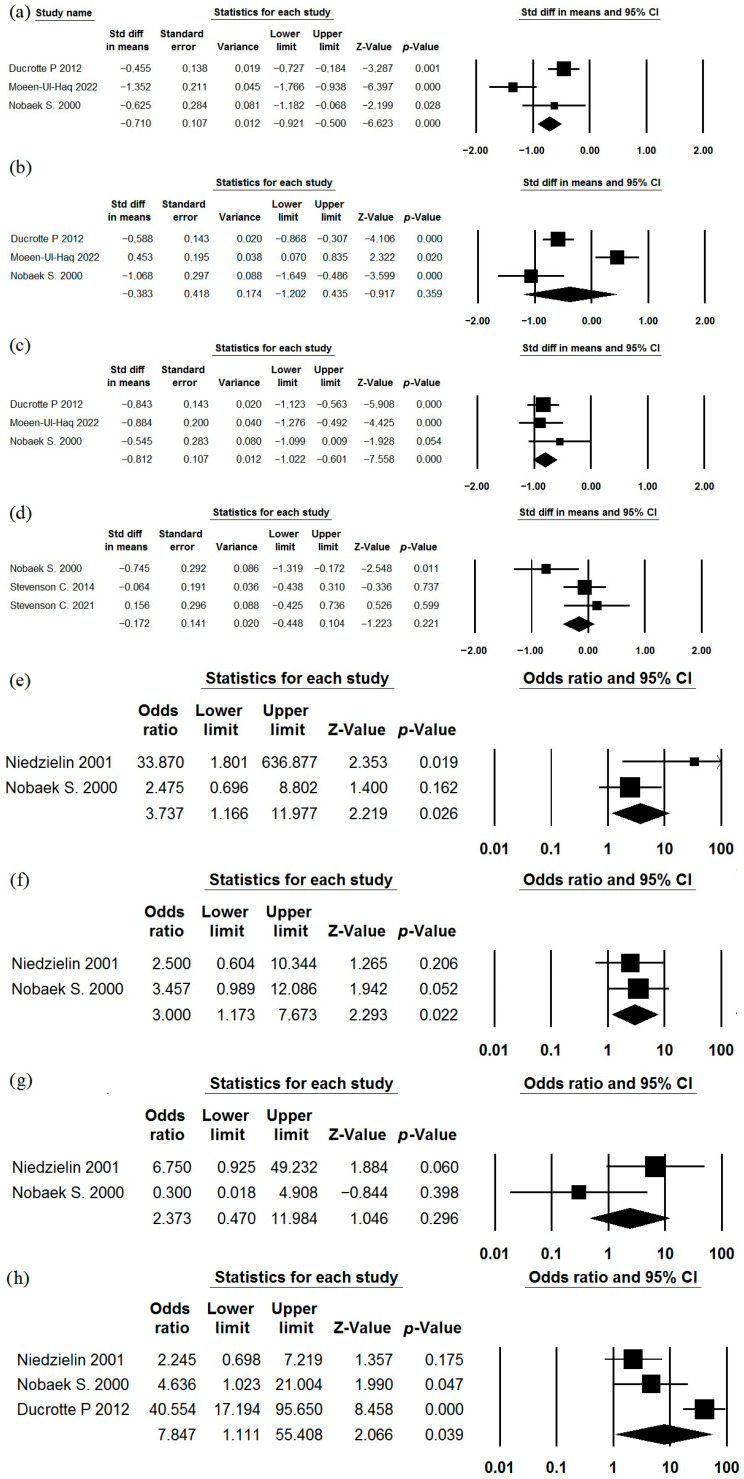
Meta-analysis of studies on the effect of *Lactobacillus plantarum* 299v on the severity of abdominal pain (**a**), bloating/flatulence (**b**), feeling of incomplete evacuation/disordered defecation function (**c**), digestive symptoms in general (**d**), abdominal pain improvement rate (**e**), flatulence improvement rate (**f**), constipation improvement rate (**g**), and rate of improvement of digestive symptoms in general (**h**) in irritable bowel syndrome.

The use of *Lactiplantibacillus plantarum* (*Lactobacillus plantarum*) 299v (DSM 9843) as a single strain has been studied in the treatment of IBS in six studies [63,64,65,66,67,68] that met the criteria (Table 1, Table 2 and Table 3). A meta-analysis of their results showed that this strain effectively reduces the severity of abdominal pain and feelings of incomplete evacuation/disordered defecation function. The number of patients reporting significant improvements in abdominal pain, flatulence, and general digestive symptoms was higher in the probiotic group than in the placebo group. This strain did not have a significant effect on the rate of constipation resolution and severity of bloating/flatulence (Figure 5). The heterogeneity of the results of studies was different (I^2^ = 50.1; 0.0; 62.9; 61.1; 0,0; 88.4; 68.4; 92.1%, respectively). The incidence of adverse effects in the probiotic groups did not differ significantly from that observed in the placebo groups (Table 2).

### 3.4. Escherichia coli Nissle 1917

**Figure 6:**
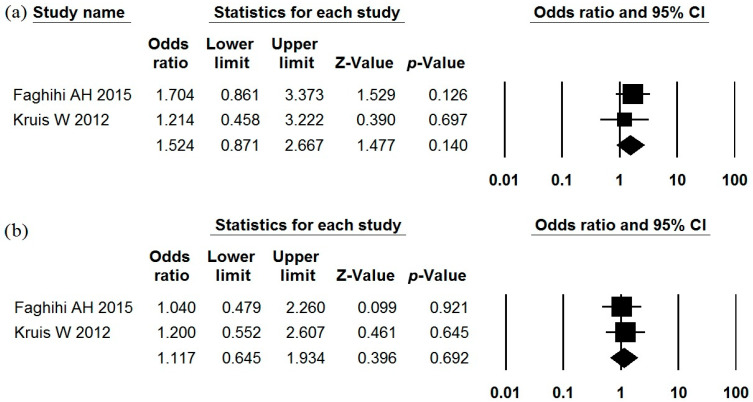
Meta-analysis of studies on the effect of *Escherichia coli* Nissle 1917 on abdominal pain improvement rate (**a**) and urgency improvement rate (**b**) in irritable bowel syndrome.

The use of *Escherichia coli* Nissle 1917 as a single-strain probiotic in the treatment of IBS was studied in two studies [69,70] that met the inclusion criteria (Table 1, Table 2 and Table 3). Of the data presented, only the incidence of reduction in abdominal pain and urgency could be included in a meta-analysis. Neither of these outcomes showed a significant difference between the probiotic and placebo groups (Figure 6). There was no heterogeneity in the results of these studies (I^2^ = 0% for both). The incidence of adverse effects in the probiotic groups did not differ significantly from that observed in the placebo groups (Table 2).

### 3.5. Saccharomyces cerevisiae CNCM I-3856

**Figure 7:**
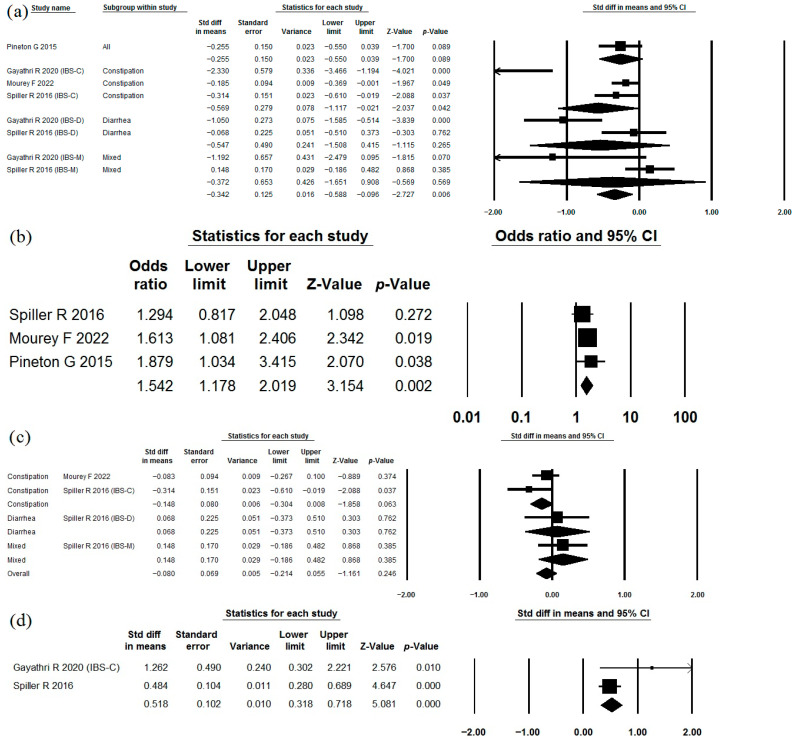
Meta-analysis of studies on the effect of *Saccharomyces cerevisiae* CNCM I-3856 on abdominal pain severity (**a**) and improvement rate (**b**), bloating severity (**c**), and Bristol Stool Scale improvement (**d**) in irritable bowel syndrome.

The use of *Saccharomyces cerevisiae* CNCM I-3856 as a single-strain probiotic in the treatment of IBS was studied in four studies [71,72,73,74] that met the criteria (Table 1, Table 2 and Table 3). Moreover, in 2 of them, data were provided separately for each form of IBS [71,74]. This strain reduced abdominal pain severity in the meta-analysis of all data, as well as in the constipation-predominant subgroups, but not in the diarrhea-predominant and mixed-disorders subgroups (Figure 7a). The heterogeneity of the study’s results was high (I^2^ = 76.9). No publication bias was detected (*p* = 0.174; Figure 4b). Patients who received this strain were more likely to report a reduction in abdominal pain than those in the placebo group (Figure 7b). This strain had no significant effect on bloating (Figure 7c; I^2^ = 35.5). This strain significantly improved stool consistency (Bristol Stool Scale) in the constipation-predominant subgroup (Figure 7d; I^2^ = 58.5). The incidence of adverse effects in the probiotic groups did not differ significantly from that observed in the placebo groups (Table 2).

### 3.6. Lactobacillus gasseri BNR17

**Figure 8:**
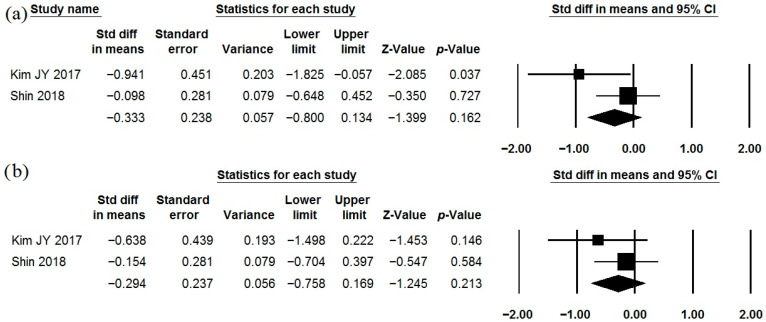
Meta-analysis of studies on the effect of *Lactobacillus gasseri* BNR17 on severity of abdominal pain (**a**) and bloating (**b**) in irritable bowel syndrome.

The effects of *Lactobacillus gasseri* BNR17 as a single-strain probiotic were described in two studies (Table 1, Table 2 and Table 3) [75,76], the data of which had to be reprocessed before meta-analysis. This strain did not show a significant effect on abdominal pain and bloating (Figure 8). The heterogeneity of the study results varied (I^2^ = 76.9 and 0.0%, respectively). Further studies with standardized outcome reporting (mean ± standard deviation or standard error of the mean before and after the study) are needed to clarify the effect of this strain on IBS symptoms. The incidence of adverse effects in the probiotic groups did not differ significantly from that observed in the placebo groups (Table 2).

### 3.7. Bacillus coagulans Unique IS2 (MTCC5260)

**Figure 9:**
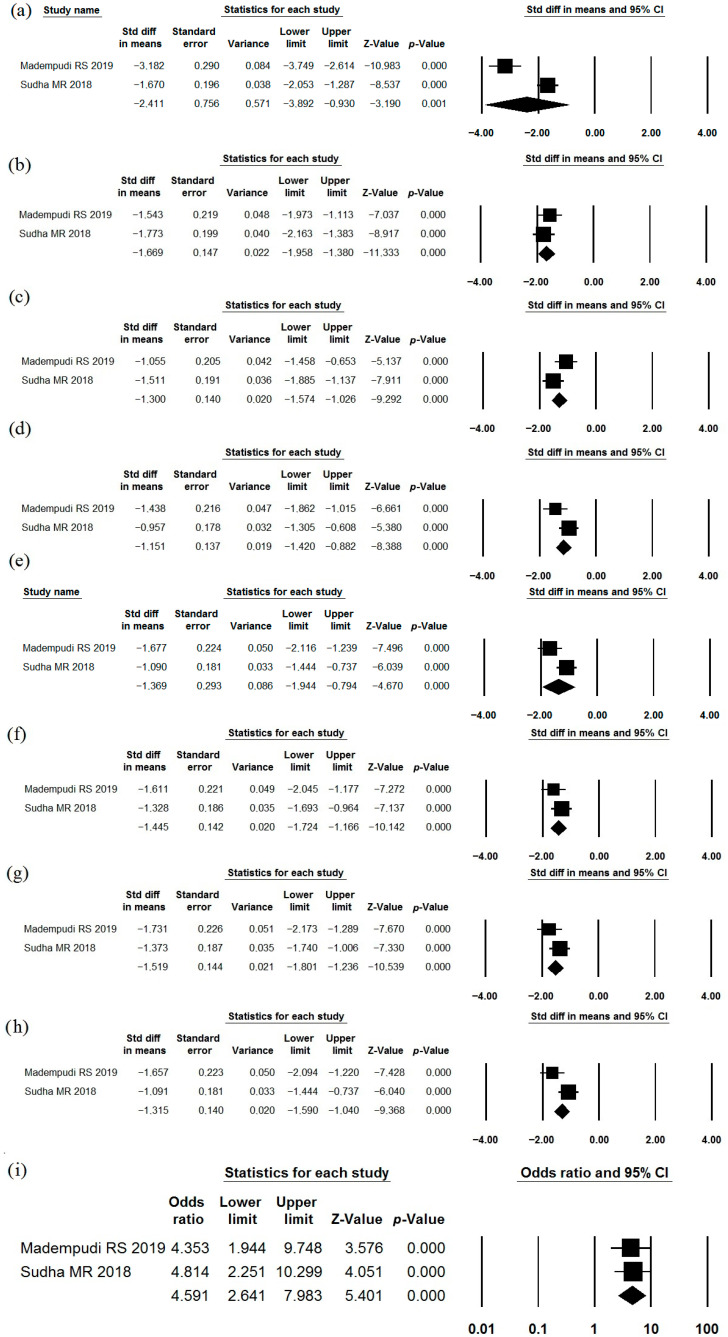
Meta-analysis of studies on the effect of *Bacillus coagulans* Unique IS2 on severity of abdominal pain (**a**), bloating (**b**), urgency (**c**), incomplete evacuation (**d**), straining (**e**), flatulence (**f**), bowel habit dissatisfaction (**g**), and IBS symptoms in general (**h**), as well as the frequency of normalization of stool consistency after taking this probiotic compared with placebo (**i**).

The effects of *Bacillus coagulans* Unique IS2 (MTCC5260) as a single-strain probiotic were described in two studies (Table 1, Table 2 and Table 3) [77,78]. This strain reduced the severity of abdominal pain, bloating, urgency, incomplete evacuation, straining, flatulence, bowel habit dissatisfaction, and overall IBS symptoms (Figure 9a–h). The heterogeneity of the study results varied (I^2^ = 94.7%; 0.0%; 64.1%; 66.3%; 76.0%; 0.0%; 33.0%; 74,3%). Normalization of stool consistency occurred more frequently than in the placebo group (Figure 9i; I^2^ = 0.0%). The incidence of adverse effects in the probiotic groups did not differ significantly from that observed in the placebo groups (Table 2).

### 3.8. Saccharomyces boulardii CNCM I-745

**Figure 10:**
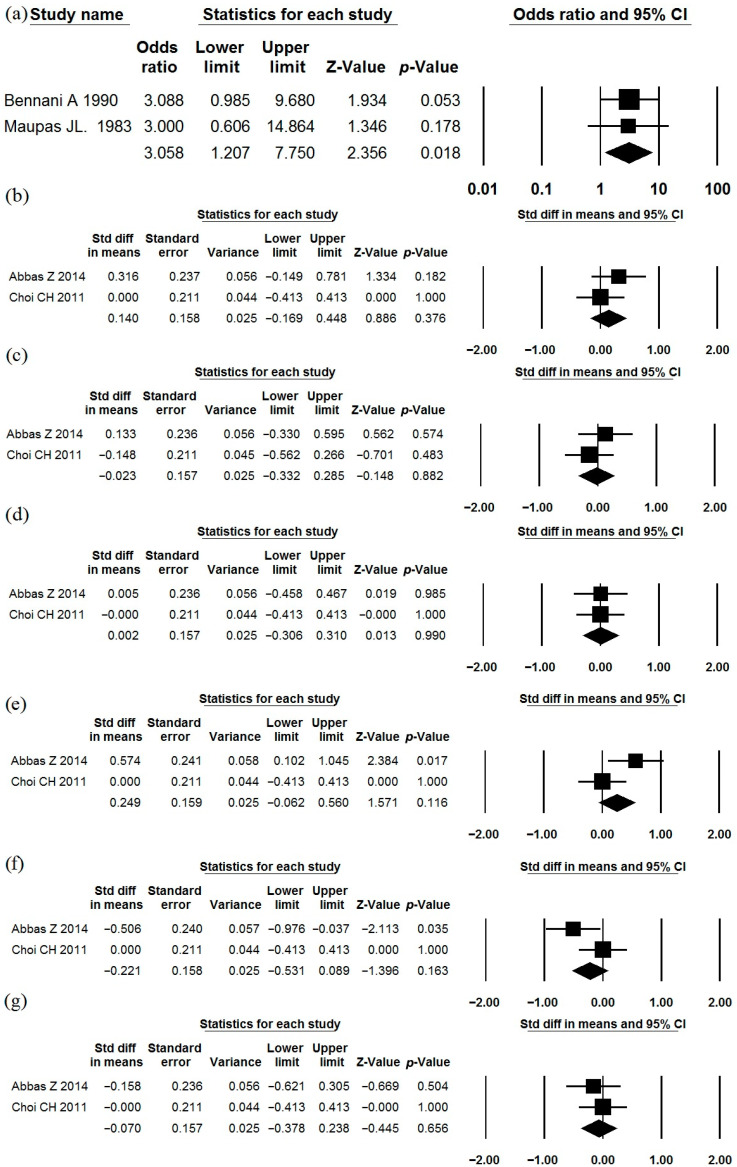
Meta-analysis of studies on the effect of *Saccharomyces boulardii* CNCM I-745 on rate of patients with significant reductions in abdominal pain (**a**), as well as on severity of abdominal pain (**b**), bloating (**c**), urgency (**d**), straining (**e**), incomplete evacuation (**f**), and passage of mucus (**g**).

The effects of *Saccharomyces boulardii* CNCM I-745 as a single-strain probiotic were described in five studies (Table 1, Table 2 and Table 3) [79,80,81,82,83]. We were unable to obtain the full text of one of these studies [83], and therefore its data were not included in our meta-analyses. In two studies, this strain was more likely to produce significant reductions in abdominal pain compared to placebo (Figure 10a; I^2^ = 0.0%). However, analysis of the other two studies, which reported the values of symptom scales before and after treatment rather than the number of patients with symptom improvement, showed that this strain did not have a sufficient effect on the severity of abdominal pain, bloating, urgency, straining, sense of incomplete evacuation, and passage of mucus (Figure 10b–g; I^2^ = 0.0%; 0.0%; 0.0%; 68.9%; 60.3%; 0.0%, respectively). These studies were characterized by high variability in the characteristics (mean values were often equal to or less than the standard deviation). Further studies are needed to clarify its effectiveness in the treatment of IBS. The incidence of adverse effects in the probiotic groups did not differ significantly from that observed in the placebo groups (Table 2).

### 3.9. Bacillus coagulans MTCC 5856

**Figure 11:**
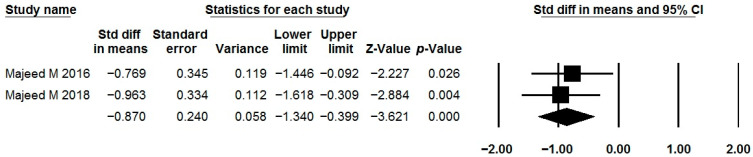
Meta-analysis of studies on the effect of *Bacillus coagulans* MTCC 5856 on quality of life of IBS patients.

The effects of *Bacillus coagulans* MTCC 5856 as a single-strain probiotic were described in two studies (Table 1, Table 2 and Table 3) [84,85]. Unfortunately, the effect of this probiotic on quality of life is the only parameter whose results could be meta-analyzed. This strain showed a positive effect on this parameter (Figure 11; I^2^ = 0.0%). The incidence of adverse effects in the probiotic groups did not differ significantly from that observed in the placebo groups (Table 2).

### 3.10. Lactobacillus casei Shirota

**Figure 12:**
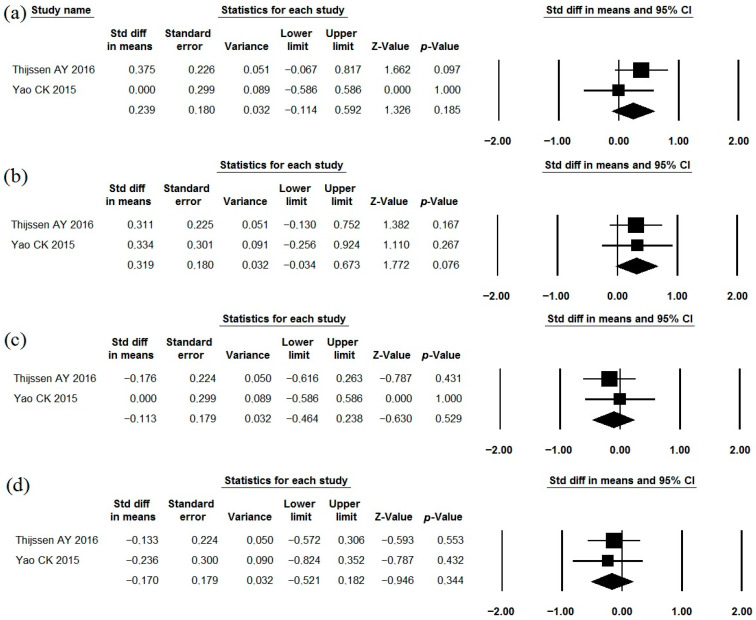
Meta-analysis of studies on the effect of *Lactobacillus casei* Shirota on severity of abdominal pain (**a**), bloating (**b**), flatulence (**c**), and IBS symptoms in general (**d**).

The effects of *Lactobacillus casei* Shirota as a single-strain probiotic were described in two studies (Table 1, Table 2 and Table 3) [86,87]. This strain did not have a sufficient effect on the severity of abdominal pain, bloating, flatulence, and IBS symptoms in general (Figure 12; I^2^ = 0.0%; 0.0%; 0.0%; 68.9%, respectively). The incidence of adverse effects in the probiotic groups did not differ significantly from that observed in the placebo groups (Table 2).

## 4. Discussion

A meta-analysis of RPCT outcomes is the gold standard for confirming its effectiveness in treating an illness. We were able to identify ten probiotic strains whose data could be meta-analyzed out of the numerous probiotics evaluated for the treatment of IBS. *Bifidobacterium longum* 35624, *Lactobacillus rhamnosus* GG, *Lactobacillus plantarum* 299v (DSM 9843), *Saccharomyces cerevisiae* CNCM I-3856, and *Bacillus coagulans* Unique IS2 were found to be associated with an improvement of all or core IBS symptoms.

The work by Hun L. et al. [88], which detailed the impact of *Bacillus coagulans* GBI-30, 6086 on IBS, was excluded from the analysis since we were unable to access the complete text of the article by Dolin BJ et al. [87] that studied the same strain.

Despite the fact that 30 systematic studies (Supplementary Table S1) on the effects of probiotics in IBS have been published thus far, the majority of them either describe all of these drugs collectively or additionally perform analysis by genus or, less frequently, species. More than four years have passed since the publication of the last systematic study that attempted to perform a strain-specific analysis [33]. We were able to considerably update the data in that review by incorporating 13 new studies into our meta-analyses. According to that review, *Bacillus coagulans* MTCC5260 and *Lactobacillus rhamnosus* GG lessen the intensity of abdominal pain in IBS, while *Bacillus coagulans* MTCC5260, *Lactobacillus plantarum* 299v, *Saccharomyces boulardii* CNCM I-745, and *Saccharomyces cerevisiae* CNCM I-3856 increase the frequency of abdominal pain relief, which is in line with our findings.

According to a systematic review by Goodoory et al. [10], *Saccharomyces cerevisiae* I-3856 significantly reduces abdominal pain, while *Lactobacillus plantarum* 299V improves overall IBS symptoms without significantly affecting abdominal pain. *Bifidobacterium bifidum* MIMBb75 has little effect on overall IBS symptoms. Bloating is likewise unaffected by the latter. These results align with our findings for *Saccharomyces cerevisiae* I-3856. Since the effects of living and dead bacteria can differ greatly, we excluded *Bifidobacterium bifidum* MIMBb75 from our evaluation because one [89] of the two studies included in that review investigated dead bacteria (metabiotics) of this strain. In contrast to that review, ours showed that *Lactobacillus plantarum* 299V reduces the intensity of IBS-related abdominal pain. Unfortunately, a specific forest plot for this strain was not included in that study, which would have helped us comprehend the variations in review outcomes. Furthermore, in contrast to that review, ours examined seven additional strains and a greater number of IBS symptoms. Consistent with our results, Wen et al. [17] demonstrated that *Lactobacillus casei* Shirota has no discernible effect on IBS. In that review, other strains were not examined independently.

Consistent with our findings, Ford et al. [20] showed that *Lactobacillus plantarum* DSM 9843 (299V) considerably decreased the intensity of IBS symptoms. In that analysis, *Bifidobacterium longum* 35624 was not found to be beneficial for stomach pain; however, our review, which included additional research, discovered that this strain was beneficial for IBS symptoms. In that review, the outcomes of other strains were not independently meta-analyzed.

In line with our findings, Wu et al. [26] showed that *Saccharomyces cerevisiae* I-3856 was useful in lessening the intensity of IBS symptoms. In that review, the outcomes of other strains were not independently meta-analyzed.

The mechanism by which probiotics may exert a beneficial effect in IBS is complex and not fully understood, much as IBS pathogenesis itself. It is believed that probiotics strengthen the intestinal barrier and modulate the composition and function of the gut microbiota, altering the signals it transmits to the intestinal nervous and immune systems and thereby reducing visceral sensitivity and normalizing intestinal motility [90,91,92,93].

A limitation of our systematic review is that we were unable to obtain the full texts of two articles [83,87], whose data could have influenced the results of our review. In addition, the lack of a standard form for reporting study results (mean and standard deviation for results before and after treatment) could have led to biased data conversion. Furthermore, most meta-analyses included only a small number of studies.

Another limitation is that a number of potentially beneficial probiotic strains were excluded from our meta-analyses because they only had one RCPI. This could lead to selection bias, favoring the most well-known probiotic strains over others that are less well-known and, hence, have received less research. Table 4 summarizes the findings of studies on the efficacy of excluded probiotic strains in the treatment of IBS [87,88,94,95,96,97,98,99,100,101,102,103,104,105,106,107,108,109,110,111,112,113,114,115,116,117,118,119,120,121,122,123].

Our research is further limited by the fact that we often meta-analyzed studies that used various approaches, such as varying probiotic dosages, treatment durations, IBS variants, participant age, and diagnostic criteria (Rome I, II, III, IV, and others). We conducted subgroup meta-analyses for various patient age groups and IBS variations in addition to the overall meta-analysis wherever feasible (e.g., for *Lactobacillus rhamnosus* GG and *Saccharomyces cerevisiae* CNCM I-3856). However, unfortunately, in most cases, the number of studies was too small to conduct subgroup analyses.

A strength of our work is that it represents the most up-to-date and most comprehensive strain-specific meta-analyses on this topic.

More strain-specific RPCTs are needed for subsequent meta-analyses with other probiotic strains tested in IBS.

## 5. Conclusions

Meta-analyses confirmed the efficacy of *Bifidobacterium longum* 35624, *Lactobacillus rhamnosus* GG, *Lactobacillus plantarum* 299v (DSM 9843), *Saccharomyces cerevisiae* CNCM I-3856, and *Bacillus coagulans* Unique IS2 in relation to the main symptoms of IBS. *Bacillus coagulans* MTCC 5856 improves quality of life for those with IBS. Conflicting results were obtained from meta-analyses of *Saccharomyces boulardii* CNCM I-745 efficacy. Meta-analyses did not confirm the efficacy of *Escherichia coli* Nissle 1917, *Lactobacillus gasseri* BNR17, and *Lactobacillus casei* Shirota in the treatment of IBS.

## Figures and Tables

**Table 1:** Main characteristics of the included studies.

Study	Country	Probiotic	Strain	Dose	Dura-Tion	Probiotic/Placebo (Pr/Pl)	Rome Criteria	IBS Pattern (D/C/M/U)	Male/ Female	Mean Age (Pr/Pl)
Charbonneau D 2013 [55]	USA	*Bifidobacterium infantis*	35624	10^9^ cells/d	8 wk	39/37	II	ND	14/62	47/43
O’Mahony L 2005 [56]	Ireland	*Bifidobacterium infantis*	35624	10^10^ cells/d	8 wk	22/22	II	21/20/34/0	27/48	44
Whorwell PJ 2006 [57]	UK	*Bifidobacterium infantis*	35624	10^8^ cells/d	4 wk	72/76	II	163/60/70/0	0/148	43/42
Bauserman M 2005 [58]	USA	*Lactobacillus rhamnosus*	GG	2 × 10^10^ cells/d	6 wk	25/25	II	ND	10/40	12/12
Francavilla R 2010 [59]	Italy	*Lactobacillus rhamnosus*	GG	6 × 10^9^ cells/d	8 wk	67/69	II	ND	78/47	7/6
Gawrońska A 2007 [60]	Poland	*Lactobacillus rhamnosus*	GG	6 × 10^9^ cells/d	4 wk	18/19	II	ND	ND	12/11
Kianifar H 2015 [61]	Iran	*Lactobacillus rhamnosus*	GG	2 × 10^10^ cells/d	4 wk	26/26	III	9/12/31/0	27/25	7/7
O’Sullivan MA 2000 [62]	Ireland	*Lactobacillus rhamnosus*	GG	4×10^10^ cells/d	4 wk	24/19	I	ND	3/16	39
Ducrotté P 2012 [63]	France+ India	*Lactobacillus plantarum*	299v	10^10^ cells/d	4 wk	105/99	III	ND	151/63	37/38
Moeen-Ul-Haq 2022 [64]	Pakistan	*Lactobacillus plantarum*	299v	5 × 10^10^ cells/d	4 wk	55/53	III	33/28/47/0	63/45	38/34
Niedzielin 2001 [65]	Poland	*Lactobacillus plantarum*	299v	2 × 10^10^ cells/d	4 wk	20/20	Manning criteria	ND	8/32	45
Nobaek S. 2000 [66]	Sweden	*Lactobacillus plantarum*	299v	2 × 10^10^ cells/d	4 wk	25/27	I	ND	16/36	51/46
Stevenson C. 2014 [67]	South Africa	*Lactobacillus plantarum*	299v	10^10^ cells/d	8 wk	23 + 17/25	II	ND	2/79	48/47
Stevenson C. 2021 [68]	South Africa	*Lactobacillus plantarum*	299v	10^10^ cells/d	8 wk	35/17	II	28/24/0/0	1/51	52/46
Faghihi AH 2015 [69]	Iran	*Escherichia coli*	Nissle 1917	ND	6 wk	67/69	II	49/55/30/0	68/71	39/38
Kruis W 2012 [70]	Germany	*Escherichia coli*	Nissle 1917	0.5–5×10^10^ cells/d	12 wk	54/49	II	35/54/71/0	28/92	46/45
Gayathri R 2020 [71]	India	*Saccharomyces cerevisiae*	CNCM I-3856	4 × 10^9^ cells/d	8 wk	48/44	III	61/20/11/0	66/34	42/40
Mourey F 2022 [72]	France	*Saccharomyces cerevisiae*	CNCM I-3856	8 × 10^9^ cells/d	8 wk	208/201	IV	0/409/0/0	86/392	41/40
Pineton G 2015 [73]	France	*Saccharomyces cerevisiae*	CNCM I-3856	8 × 10^9^ cells/d	8 wk	86/93	III	51/84/44/0	25/154	43/45
Spiller R 2016 [74]	France	*Saccharomyces cerevisiae*	CNCM I-3856	8 × 10^9^ cells/d	12 wk	132/131	III	79/180/120/0	62/317	45/45
Kim JY 2017 [75]	Korea	*Lactobacillus gasseri*	BNR17	10 × 10^9^ cells/d	4 wk	10/12	ND	ND	13/9	29/27
Shin 2018 [76]	Korea	*Lactobacillus gasseri*	BNR17	10 × 10^9^ cells/d	8 wk	24/27	III	51/0/0/0	22/29	35/38
Madempudi RS 2019 [77]	India	*Bacillus coagulans*	Unique IS2	2 × 10^9^ cells/d	8 wk	53/55	III	3/35/70/0	78/30	44/42
Sudha MR 2018 [78]	India	*Bacillus coagulans*	Unique IS2	ND	8 wk	72/69	III	58/73/10/0	80/61	8/8
Bennani A 1990 [79]	France	*Saccharomyces boulardii*	CNCM I-745	500 mg/d	30 days	28/34	ND	27/10/18/0	19/43	35
Maupas JL. 1983 [80]	France	*Saccharomyces boulardii*	CNCM I-745	9 caps/d	1 mo	16/18	ND	ND	20/14	42
Abbas Z 2014 [81]	Pakistan	*Saccharomyces boulardii*	CNCM I-745	750 mg/d	6 wk	37/35	III	72/0/0/0	53/19	38/33
Choi CH 2011 [82]	Korea	*Saccharomyces boulardii*	CNCM I-745	8×10^11^ cells/d	4 wk	45/45	II	53/0/21/0	37/37	40/41
Majeed M 2016 [83]	India	*Bacillus coagulans*	MTCC 5856	2 × 10^9^ cells/d	90 days	18/18	III	36/0/0/0	17/19	36/35
Majeed M 2018 [84]	India	*Bacillus coagulans*	MTCC 5856	2 × 10^9^ cells/d	90 days	20/20	III	ND	6/34	40/44
Thijssen AY 2016 [85]	Netherlands	*Lactobacillus casei*	Shirota	6.5 × 10^9^ cells/d	8 wk	39/41	II	24/20/23/13	25/55	41/42
Yao CK 2015 [86]	Australia	*Lactobacillus casei*	Shirota	6.5 × 10^9^ cells/d	6 wk	24/21	III	17/17/15/7	10/46	41/31

Note: ND—no data.

**Table 2:** Parameters reported in the included studies.

Study	Symptom Scale	IBS Symptoms in General	Abdominal Pain	Bloating	Urgency	Straining	Incomplete Evacuation	Passage of Mucus	Flatulence	Stool Consistency	Number of Bowel Habits	QoL Scale	Adverse Effects
Charbonneau D 2013	6-point scale	+	+	+	+	+	+		+	+	+		NSD
O’Mahony L 2005	7-point Likert scale	+	+	+		+						IBS-QOL	NSD
Whorwell PJ 2006	6-point scale	+	+	+	+	+	+		+		DCnMA		NSD
Bauserman M 2005	GSRS		+										ND
Francavilla R 2010	11-point VAS		+										NAE
Gawrońska A 2007	Faces Pain Scale		+										NAE
Kianifar H 2015	5-point Likert scale		+								+		NAE
O’Sullivan MA 2000	5-point Likert scale		+	+	+						DCnMA		NSD
Ducrotté P 2012	4-point scale	CA	+	+			+				+		NSD
Moeen-Ul-Haq 2022	11-point VAS		+	+			+						ND
Niedzielin 2001	11-point scale	CA	CA	CA							CA		NAE
Nobaek S. 2000	8-point VAS	+	+/CA	+/CA			+			+	CA		NAE
Stevenson C. 2014	FS-IBS-S	+										QoL-IBS	NSD
Stevenson C. 2021	FS-IBS-S	+											NSD
Faghihi AH 2015	B-IBS-SQ	+	CA		+/CA	CA	CA	CA		CA			ND
Kruis W 2012	IMPSS	CA	CA	CA	CA				CA			HRQL	NSD
Gayathri R 2020	8-point Likert scale		+/CA							+			NSD
Mourey F 2022	8-point Likert scale		+/CA	+								IBS-QOL	NSD
Pineton G 2015	8-point Likert scale		+/CA										NSD
Spiller R 2016	8-point Likert scale	+/CA	+	+	+				+	+		IBS-QOL	NSD
Kim JY 2017	5-point Likert scale		+	+			+						ND
Shin 2018	6-point scale	+	+	+						+/CA		IBS-QOL	NSD
Madempudi RS 2019	11-point scale	+/CA	+/CA	+	+	+	+		+	CA	+		NAE
Sudha MR 2018	11-point Likert scale	+	+/CA	+	+	+	+		+	CA			NSD
Bennani A 1990	4-point scale	DCnMA	CA	CA						CA			NAE
Maupas JL. 1983	5-point scale	DCnMA	CA	DCnMA							DCnMA		NAE
Abbas Z 2014	4-point scale	+	+	+	+	+	+	+	+	+	+	IBS-QOL	NSD
Choi CH 2011	7-point Likert scale	+	+	+	+	+	+	+	+	+	+	IBS-QOL	NSD
Majeed M 2016	VAS		+	+						+		IBS-QOL	NSD
Majeed M 2018	Not assessed											IBS-QOL	NSD
Thijssen AY 2016	5-point Likert scale	+	+	+					+			RAND-36	NSD
Yao CK 2015	VAS	+	+	+					+				NSD

Note: B-IBS-SQ—Birmingham IBS Symptom Questionnaire; CA—categorical assessment (improvement/non-improvement); DCnMA—data cannot be meta-analyzed; IMPSS—Integrative Medicine Patient Satisfaction Scale; FS-IBS-S—Francis Severity IBS score; NAE—no adverse effects; ND—no data; NSD—no significant difference between probiotic and placebo groups; QoL—quality of life; VAS—visual analog scale.

**Table 3:** Assessment of biases in included studies.

Study	R	D	Mi	Me	S	O
Charbonneau D 2013	+	+	+	+	+	+
O’Mahony L 2005	?	?	?	+	+	?
Whorwell PJ 2006	+	+	+	+	+	+
Bauserman M 2005	+	+	+	?	+	+
Francavilla R 2010	+	+	+	+	+	+
Gawrońska A 2007	+	+	+	+	+	+
Kianifar H 2015	?	+	+	+	+	+
O’Sullivan MA 2000	?	+	?	?	+	?
Ducrotté P 2012	+	+	+	+	+	+
Moeen-Ul-Haq 2022	+	+	+	+	+	+
Niedzielin 2001	?	?	+	+	+	+
Nobaek S. 2000	+	+	+	+	+	+
Stevenson C 2014	+	+	+	+	+	+
Stevenson C. 2021	+	+	+	+	+	+
Faghihi AH 2015	+	+	?	?	+	+
Kruis W 2012	+	+	+	?	+	+
Gayathri R 2020	?	?	+	+	+	+
Mourey F 2022	+	+	+	+	+	+
Pineton G 2015	+	+	?	+	+	+
Spiller R 2016	+	+	+	+	+	+
Kim JY 2017	+	+	?	?	+	+
Shin 2018	?	+	?	?	+	?
Madempudi RS 2019	+	+	+	+	+	+
Sudha MR 2018	+	+	+	+	+	+
Bennani A 1990	+	+	+	+	+	+
Maupas JL. 1983	+	+	?	?	+	+
Abbas Z 2014	+	+	+	+	+	+
Choi CH 2011	+	+	+	+	+	+
Majeed M 2016	+	+	+	+	+	+
Majeed M 2018	+	+	+	+	+	+
Thijssen AY 2016	+	+	+	+	+	+
Yao CK 2015	?	+	+	+	+	+

Note: green colour and “+” sign—low bias risk, yellow and “?” sign—moderate bias risk.

**Table 4:** Main results of efficacy of excluded probiotic strains in the treatment of irritable bowel syndrome (IBS).

Study	Probiotic	Strain	Main Effects
Skrzydło-Radomańska 2023 [98]	*Bacillus coagulans*	BC300 (DSM 33836)	Pr reduced some IBS symptoms
Shaikh 2024 [99]	*Bacillus coagulans*	BCP92	Pr reduced IBS severity
Dolin 2009 [87]	*Bacillus coagulans*	GBI-30, 608	Pr reduced number of bowel movements
Hum 2009 [88]	*Bacillus coagulans*	GBI-30, 608	Pr reduced severity of abdominal pain and bloating
Gupta 2021 [100]	*Bacillus coagulans*	LBSC (DSM17654)	Pr reduced bloating, abdominal pain, diarrhea, constipation, stomach rumbling, nausea, vomiting, headache, and anxiety
Kallur 2024 [101]	*Bacillus coagulans*	LMG S-31876	Pr reduced abdominal pain, bloating, and diarrhea
Garvey 2022 [102]	*Bacillus subtilis*	BS50	Pr reduced bloating, burping, and flatulence
Guglielmetti 2011 [104]	*Bifidobacterium bifidum*	MIMBb75 (SYN-HI-001)	Pr reduced global IBS symptoms, abdominal pain, bloating, and improved quality of life
Skrzydło-Radomańska 2023 [98]	*Bifidobacterium lactis*	BI040 (DSM 33812/34614)	Pr reduced some IBS symptoms
JanssenDuijghuijsen 2024 [103]	*Bifidobacterium lactis*	BLa80	Pr reduced IBS severity
Martoni 2020 [108]	*Bifidobacterium lactis*	UABla-12	Pr reduced abdominal pain and abdominal distension, normalized bowel habits and improved quality of life
Srivastava 2024 [106]	*Bifidobacterium longum*	CECT 7347	Pr reduced IBS severity and improved quality of life
Pinto-Sanchez 2017 [95]	*Bifidobacterium longum*	NCC3001	Pr increases quality of life; no significant effect on IBS symptoms
Lewis 2020 [105]	*Bifidobacterium longum*	R0175	Pr reduced IBS severity and improved quality of life
Enck 2009 [109]	*Escherichia coli*	DSM17252	Pr reduced IBS severity
Kwon 2024 [110]	*Lacticaseibacillus* (*Lactobacillus*) *rhamnosus*	IDCC 3201 (RH 3201)	Pr reduced IBS severity
Dapoigny 2012 [118]	*Lacticaseibacillus* (*Lactobacillus*) *rhamnosus*	LCR35	Pr had no effect in the whole IBS population
Jung 2022 [111]	*Lactiplantibacillus* (*Lactobacillus*) *plantarum*	APsulloc 331261 (GTB1)	Pr reduced abdominal pain, bloating, feeling of incomplete evacuation, diarrhea, and improved quality of life.
Yang 2021 [114]	*Lactiplantibacillus* (*Lactobacillus*) *plantarum*	CCFM1143	Pr reduced IBS severity and improved quality of life
Liu 2021 [121]	*Lactiplantibacillus* (*Lactobacillus*) *plantarum*	CCFM8610	Pr reduced IBS severity and improved quality of life
Kim 2024 [113]	*Lactiplantibacillus* (*Lactobacillus*) *plantarum*	JSA22	Pr reduced bloating
Martoni 2023 [112]	*Lactiplantibacillus* (*Lactobacillus*) *plantarum*	Lpla33 (DSM34428)	Pr reduced IBS severity
Ligaarden 2010 [96,97]	*Lactiplantibacillus* (*Lactobacillus*) *plantarum*	MF 1298	Pr had unfavorable effect on IBS symptoms
Lyra 2016 [115]	*Lactobacillus acidophilus*	NCFM	Pr reduced moderate to severe abdominal pain, but not other IBS symptoms
Sinn 2008 [116]	*Lactobacillus acidophilus*	SDC 2012, 2013	Pr reduced abdominal pain
Martoni 2020 [108]	*Lactobacillus acidophilus*	DDS-1	Pr reduced abdominal pain and abdominal distension, normalized bowel habits and improved quality of life
Murakami 2012 [117]	*Lactobacillus brevis*	KB290	Pr reduced abdominal pain
Nobutani 2017 [119]	*Lactobacillus gasseri*	CP2305	Pr reduced IBS severity
O’Mahony 2005 [56]	*Lactobacillus salivarius*	UCC4331	Pr had minimal effect on IBS symptoms
Cremon 2018 [120]	*Lactocaseibacillus* (*Lactobacillus*) *paracasei*	CNCM I-1572	Pr had no significant effect on IBS symptoms
Lewis 2020 [105]	*Lactocaseibacillus* (*Lactobacillus*) *paracasei*	HA-196	Pr reduced IBS severity and improved quality of life
Lin 2024 [122]	*Lactocaseibacillus* (*Lactobacillus*) *paracasei*	NTU 101	Pr improved peristalsis and shortened defecation interval
Niv 2005 [107]	*Limosilactobacillus* (*Lactobacillus*) *reuteri*	ATCC 55730	Pr had no significant effect on IBS symptoms
Konig 2024 [123]	*Limosilactobacillus* (*Lactobacillus*) *reuteri*	ATCC PTA 6475	Pr had no significant effect on IBS symptoms
Amirimani 2013 [94]	*Limosilactobacillus* (*Lactobacillus*) *reuteri*	DSM 17938	No significant effect on abdominal pain, bloating, and other IBS symptoms

Note: Pr—probiotic.

## Data Availability

The original contributions presented in the study are included in the Supplementary Material; further inquiries can be directed to the corresponding author.

## Supplementary Materials

The following supporting information can be downloaded at https://www.mdpi.com/article/10.3390/jcm15031152/s1. Figure S1: Subgroup meta-analysis of *Lactobacillus rhamnosus* GG’s impact on the intensity of abdominal pain in irritable bowel syndrome based on specific Rome criteria for diagnosis (**a**), day dosage (billions cells/day; **b**), and treatment duration (weeks; **c**). Table S1: Main results and limitations of recent meta-analyses of probiotic use in the treatment of irritable bowel syndrome. Table S2: PRISMA Checklist [124].

## Abbreviations

The following abbreviations are used in this manuscript:

IBS	Irritable bowel syndrome
RPCT	Randomized placebo-controlled trials
