# Supplementary material for: Strain-Specific Systematic Review with Meta-Analysis of Probiotics Efficacy in the Treatment of Irritable Bowel Syndrome
Source: J Clin Med. 2026 Feb 2;15(3):1152. doi: 10.3390/jcm15031152 (PMC12898053; doi:10.3390/jcm15031152)
Supplement: Supplementary file 1 [file jcm-15-01152-s001.zip › jcm-4099941-supplementary.pdf]

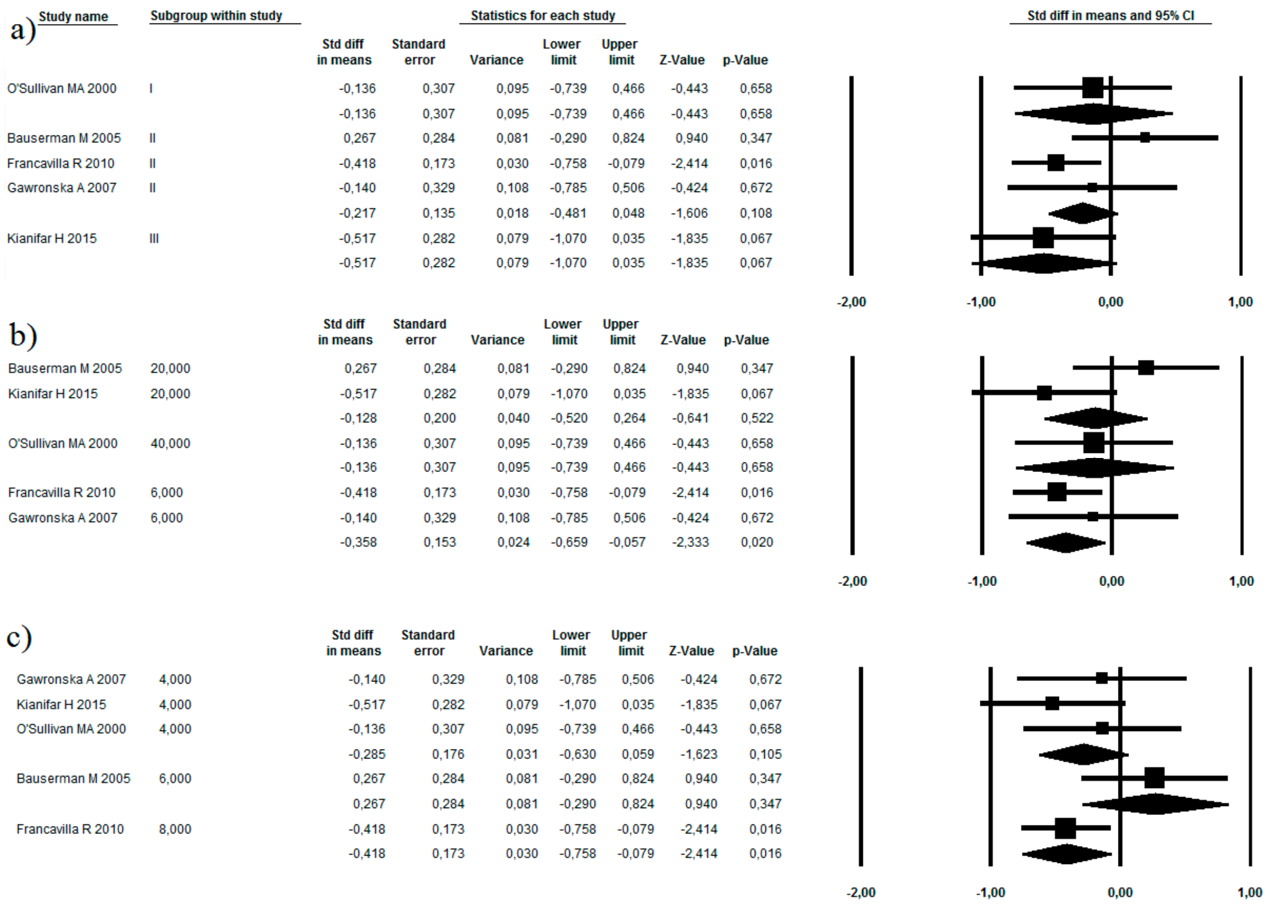


**Supplementary Figure S1.** Subgroup meta-analysis of *Lactobacillus rhamnosus GG's* impact on the intensity of abdominal pain in irritable bowel syndrome based on specific Rome criteria for diagnosis (a), day dosage (billions cells/day; b), and treatment duration (weeks; c).

**Supplementary Table S1.** Main results and limitations of recent meta-analyses of probiotic use in the treatment of irritable bowel syndrome

| Meta-analysis | Date of data search | Number of included studies | Main results | Limitations |
|---|---|---|---|---|
| Xie 2023 [9] | June 2023 | 81 | Certain probiotic strains and multi-strain formulations have been shown to be effective in treating specific manifestations of IBS | Taking into account the peculiarities of the scales for assessing the severity of IBS leads to the fact that it is not possible to summarize the data on strains whose effectiveness was studied using different scales. |
| Goodoory 2023 [10] | March 2023 | 82 | Certain types of probiotic organisms have been shown to be effective in treating specific manifestations of IBS | Most of the findings are not strain specific |
| Zhang 2023 [11] | August 2023 | 52 | Probiotics have a therapeutic role in IBS. However, the effect of different probiotics varies | No strain specificity |
| Asha 2020 [12] | June 2019 | 33 | Probiotics are generally effective for IBS, but results are highly heterogeneous | No strain specificity, more than 6 years have passed since the analysis |
| Zhang 2022[13] | August 2021 | 43 | Certain species of probiotic organisms showed to be effective in IBS | No strain specificity, more than 4 years have passed since the analysis |
| Liang 2019 [14] | April 2019 | 14 | Probiotics are generally effective for IBS | Few studies, no strain specificity, more than 6 years have passed since the analysis |
| Zhang 2016 [15] | October 2015 | 21 | Probiotics are generally effective for IBS | Few studies, no strain specificity, more than 10 years have passed since the analysis |
| Niu 2020 [16] | April 2019 | 35 | Multi-strain probiotics can improve IBS symptoms. Further research is required | No strain specificity, more than 6 years have passed since the analysis |
| Wen 2020 [17] | May 2019 | 17 | Probiotics may improve gut transit time, stool frequency, and stool consistency. RCTs are | Few studies, no strain specificity, more than 6 years have passed since the analysis |

| | | | | |
|---|---|---|---|---|
| | | | required to better determine the species or strains. | |
| Ceccherini 2022 [18] | December 2021 | 104 | *Lactobacillus rhamnosus* and *L. acidophilus* have the greatest effectiveness, especially in terms of quality of life, bloating and abdominal pain | No strain specificity, more than 3.5 years have passed since the analysis |
| Chen 2023 [19] | November 2022 | 72 | Probiotics are generally effective for IBS | No strain specificity |
| Ford 2018 [20] | July 2017 | 53 | Certain combinations of probiotics or specific species and strains appear to have a beneficial effect on overall IBS symptoms and abdominal pain, but it is not possible to draw definitive conclusions about their effectiveness | More than 8 years have passed since the analysis |
| de Sequeira 2021 [21] | April 2021 | 35 | Probiotics improve quality of life in IBS without significantly affecting anxiety and depression | No strain specificity, more than 4 years have passed since the analysis |
| Qing 2023 [22] | June 2022 | 7 | Saccharomyces reduces the severity of abdominal pain in IBS | Only probiotic fungi were analyzed |
| van der Geest 2022 [23] | January 2021 | 13 | Probiotics are generally effective for IBS. New RPCTs are required | Few studies, no strain specificity, more than 4 years have passed since the analysis |
| Xie 2022 [24] | January 2021 | 76 | *Lactobacillus* was found to be the most effective in IBS; *Bifidobacterium* and *Bacillus* may also be effective but require further confirmation | No strain specificity, more than 4 years have passed since the analysis |
| Konstantis 2023 [25] | January 2023 | 6 | Probiotics showed a beneficial effect on pain and bloating in IBS, but due to significant heterogeneity and confounding factors definitive statements cannot be made | Few studies, no strain specificity |
| Wu 2024 [26] | February 2023 | 54 | Probiotics are effective in IBS, the most effective probiotic combination or strain remains unclean | No strain specificity |
| Yang 2024 [27] | June 2023 | 20 | Probiotics in general is effective in IBS, definite conclusions are challenging due to the high heterogeneity | Few studies, no strain specificity |
| Li 2020 [28] | January 2019 | 59 | Probiotics in general is effective in IBS. | No strain specificity, more than 6 years have passed since the analysis |
| Shang 2022 [29] | March 2022 | 10 | Probiotics are generally effective for IBS with constipation, but the evidence is weak. | Few studies, no strain specificity, more than 3 years have passed since the analysis |
| Sun 2020 [30] | February 2019 | 28 | Particular combinations, species or strains of probiotics are effective for overall IBS symptoms, it is hard to derive a definite conclusion due to high heterogeneity and unclear risk of bias of some trials | No strain specificity, more than 6 years have passed since the analysis |
| Almabruk 2024 [31] | 2018 | 23 | Probiotics are generally effective in IBS, but results are highly heterogeneous | Few studies, no strain specificity, more than 6 years have passed since the analysis |
| Wang 2022 [32] | August 2021 | 10 | Very low certainty evidence showed that probiotics might be an effective in diarrhea-predominant IBS | Few studies, no strain specificity, more than 4 years have passed since the analysis |
| McFarland 2021 [33] | June 2021 | 40 | Four probiotics showed significant reductions in abdominal pain: *Bacillus coagulans*, *Lactobacillus plantarum* 299v, *Saccharomyces boulardii* CNCM I-745 and *Saccharomyces cerevisiae* CNCM I-3856 | More than 4 years have passed since the data search. |
| Lei 2025 [34] | July 2023 | 44 | Probiotics in general is effective in IBS. | No strain specificity. More emphasis on diet. |
| Anwar 2025 [35] | June 2024 | 12 | Multistrain probiotics reduced IBS-SSS and its subscores. | Only multistrain probiotics were analyzed |
| Zeraattalab-Motlagh 2025 [36] | June 2023 | 175 | Probiotics and a low-FODMAP diet, considering limitations like short-term study duration, there was an influential clinical impact | No strain specificity. Included all nutritional intervention |
| Rokkas 2026 [37] | 2024 | >9 | Some probiotics or combinations may offer benefits for patients with IBS in improving quality of life, depression, and anxiety; additional research is necessary | No strain specificity |
| Yu 2025 [38] | 2024 | 37 | Combination of trimebutine and probiotics is more effective in the treatment of IBS compared with trimebutine alone. | Only combinations of probiotics with trimebutine were analyzed |

**Supplementary Table S2.** Preferred Reporting Items for Systematic reviews and Meta-Analyses extension for Scoping Reviews (PRISMA-ScR) Checklist

| SECTION | ITEM | PRISMA-ScR CHECKLIST ITEM | REPORTED ON PAGE # |
|---|---|---|---|
| **TITLE** | | | |
| Title | 1 | Identify the report as a scoping review. | 1 |
| **ABSTRACT** | | | |
| Structured summary | 2 | Provide a structured summary that includes (as applicable): background, objectives, eligibility criteria, sources of evidence, charting methods, results, and conclusions that relate to the review questions and objectives. | 1 |
| **INTRODUCTION** | | | |
| Rationale | 3 | Describe the rationale for the review in the context of what is already known. Explain why the review questions/objectives lend themselves to a scoping review approach. | 2 |
| Objectives | 4 | Provide an explicit statement of the questions and objectives being addressed with reference to their key elements (e.g., population or participants, concepts, and context) or other relevant key elements used to conceptualize the review questions and/or objectives. | 2 |
| **METHODS** | | | |
| Protocol and registration | 5 | Indicate whether a review protocol exists; state if and where it can be accessed (e.g., a Web address); and if available, provide registration information, including the registration number. | 2 |
| Eligibility criteria | 6 | Specify characteristics of the sources of evidence used as eligibility criteria (e.g., years considered, language, and publication status), and provide a rationale. | 2 |
| Information sources* | 7 | Describe all information sources in the search (e.g., databases with dates of coverage and contact with authors to identify additional sources), as well as the date the most recent search was executed. | 2 |
| Search | 8 | Present the full electronic search strategy for at least 1 database, including any limits used, such that it could be repeated. | 2 |
| Selection of sources of evidence† | 9 | State the process for selecting sources of evidence (i.e., screening and eligibility) included in the scoping review. | 2 |
| Data charting process‡ | 10 | Describe the methods of charting data from the included sources of evidence (e.g., calibrated forms or forms that have been tested by the team before their use, and whether data charting was done independently or in duplicate) and any processes for obtaining and confirming data from investigators. | 3 |
| Data items | 11 | List and define all variables for which data were sought and any assumptions and simplifications made. | 3 |
| Critical appraisal of individual sources of evidence§ | 12 | If done, provide a rationale for conducting a critical appraisal of included sources of evidence; describe the methods used and how this information was used in any data synthesis (if appropriate). | 3 |
| Synthesis of results | 13 | Describe the methods of handling and summarizing the data that were charted. | 3 |
| **RESULTS** | | | |
| Selection of sources of evidence | 14 | Give numbers of sources of evidence screened, assessed for eligibility, and included in the review, with reasons for exclusions at each stage, ideally using a flow diagram. | 2 |
| Characteristics of sources of evidence | 15 | For each source of evidence, present characteristics for which data were charted and provide the citations. | 4 |

| SECTION | ITEM | PRISMA-ScR CHECKLIST ITEM | REPORTED ON PAGE # |
|---|---|---|---|
| Critical appraisal within sources of evidence | 16 | If done, present data on critical appraisal of included sources of evidence (see item 12). | 6 |
| Results of individual sources of evidence | 17 | For each included source of evidence, present the relevant data that were charted that relate to the review questions and objectives. | 4-6 |
| Synthesis of results | 18 | Summarize and/or present the charting results as they relate to the review questions and objectives. | 6-16 |
| **DISCUSSION** | | | |
| Summary of evidence | 19 | Summarize the main results (including an overview of concepts, themes, and types of evidence available), link to the review questions and objectives, and consider the relevance to key groups. | 14 |
| Limitations | 20 | Discuss the limitations of the scoping review process. | 17 |
| Conclusions | 21 | Provide a general interpretation of the results with respect to the review questions and objectives, as well as potential implications and/or next steps. | 17-18 |
| **FUNDING** | | | |
| Funding | 22 | Describe sources of funding for the included sources of evidence, as well as sources of funding for the scoping review. Describe the role of the funders of the scoping review. | 3 |

JBI = Joanna Briggs Institute; PRISMA-ScR = Preferred Reporting Items for Systematic reviews and Meta-Analyses extension for Scoping Reviews.
* Where *sources of evidence* (see second footnote) are compiled from, such as bibliographic databases, social media platforms, and Web sites.
† A more inclusive/heterogeneous term used to account for the different types of evidence or data sources (e.g., quantitative and/or qualitative research, expert opinion, and policy documents) that may be eligible in a scoping review as opposed to only studies. This is not to be confused with *information sources* (see first footnote).
‡ The frameworks by Arksey and O'Malley (6) and Levac and colleagues (7) and the JBI guidance (4, 5) refer to the process of data extraction in a scoping review as data charting.
§ The process of systematically examining research evidence to assess its validity, results, and relevance before using it to inform a decision. This term is used for items 12 and 19 instead of "risk of bias" (which is more applicable to systematic reviews of interventions) to include and acknowledge the various sources of evidence that may be used in a scoping review (e.g., quantitative and/or qualitative research, expert opinion, and policy document).

*From:* Tricco AC, Lillie E, Zarin W, O'Brien KK, Colquhoun H, Levac D, et al. PRISMA Extension for Scoping Reviews (PRISMAScR): Checklist and Explanation. Ann Intern Med. 2018;169:467–473. doi: 10.7326/M18-0850.
